# Association of Day Length and Weather Conditions with Physical Activity Levels in Older Community Dwelling People

**DOI:** 10.1371/journal.pone.0085331

**Published:** 2014-01-30

**Authors:** Miles D. Witham, Peter T. Donnan, Thenmalar Vadiveloo, Falko F. Sniehotta, Iain K. Crombie, Zhiqiang Feng, Marion E. T. McMurdo

**Affiliations:** 1 Ageing and Health, University of Dundee, Dundee, Scotland; 2 Dundee Epidemiology and Biostatistics Unit, University of Dundee, Dundee, Scotland; 3 Department of Health Psychology, University of Newcastle, Newcastle upon Tyne, England; 4 Department of Public Health, University of Dundee, Dundee, Scotland; 5 Department of Geography and Sustainable Development, University of St Andrews, St Andrews, Scotland; Marienhospital Herne - University of Bochum, Germany

## Abstract

**Background:**

Weather is a potentially important determinant of physical activity. Little work has been done examining the relationship between weather and physical activity, and potential modifiers of any relationship in older people. We therefore examined the relationship between weather and physical activity in a cohort of older community-dwelling people.

**Methods:**

We analysed prospectively collected cross-sectional activity data from community-dwelling people aged 65 and over in the Physical Activity Cohort Scotland. We correlated seven day triaxial accelerometry data with daily weather data (temperature, day length, sunshine, snow, rain), and a series of potential effect modifiers were tested in mixed models: environmental variables (urban vs rural dwelling, percentage of green space), psychological variables (anxiety, depression, perceived behavioural control), social variables (number of close contacts) and health status measured using the SF-36 questionnaire.

**Results:**

547 participants, mean age 78.5 years, were included in this analysis. Higher minimum daily temperature and longer day length were associated with higher activity levels; these associations remained robust to adjustment for other significant associates of activity: age, perceived behavioural control, number of social contacts and physical function. Of the potential effect modifier variables, only urban vs rural dwelling and the SF-36 measure of social functioning enhanced the association between day length and activity; no variable modified the association between minimum temperature and activity.

**Conclusions:**

In older community dwelling people, minimum temperature and day length were associated with objectively measured activity. There was little evidence for moderation of these associations through potentially modifiable health, environmental, social or psychological variables.

## Introduction

Physical activity is a key determinant of future health and disability, in older people as well as in younger people [1]. Given the considerable burden of disability and disease suffered by older people, efforts to avoid or ameliorate this burden are of major public health importance [2]. However, few older people achieve recommended levels of physical activity [3], and the search for effective ways to improve everyday activity levels in older people has not been successful to date [4]. New approaches are therefore needed to increase physical activity in older people, and for this to happen, a more complete understanding of the factors that influence physical activity levels in older people is required.

One area that has received comparatively little attention to date is the effect of environmental variables on activity levels in older people. Weather and day length are such variables, and whilst these variables themselves are not modifiable, a better understanding of their influence on activity levels would assist in finding ways to combat any deleterious effect that they might have on boosting activity levels, and might allow strategies to enhance any beneficial influence that weather and day length might have.

Weather (e.g. rainfall and temperature), day length and season have consistently been shown to affect physical activity levels in children and younger to middle aged adults [5]–[8], but less work has been done in older populations. Previous studies in functionally impaired older people have suggested that day length, maximum daily temperature and hours of sunshine are all independent predictors of activity levels as measured objectively by accelerometry [9]. There is little information available however on the influence of these variables on activity levels in an unselected older population; other previous studies showing an influence of weather variables on physical activity have focused on older people with arthritis [10], and on selected healthy older Japanese and Canadian people [11], [12]. Studies to date have not explored the effect of potential interacting factors such as age, mood, physical geography and baseline physical function.

We hypothesised that inclement weather conditions would be associated with lower objectively measured physical activity levels, and that this association would be modified by environmental, physical and psychological influences. This analysis therefore tested the effect of day length and weather conditions on objectively measured activity levels in community-dwelling older people, using data from the Physical Activity Cohort Scotland (PACS).

## Methods

### Participant Selection

The PACS cohort consists of 584 people aged 65 and over, resident in the community in Tayside, Scotland. Cohort participants were recruited from 17 primary care practices; practices were selected to give a range of rural versus urban and affluent vs deprived neighbourhoods. Sampling was stratified according to age (65–80 and 80+ years) and deprivation (Scottish Index of Multiple Deprivation score decile 1–4 vs 5–10). Within each stratum, participants were selected from a patient list within each practice via computer-generated random number generation. Potential participants were excluded from the cohort if they were resident in institutional care, unwilling to participant, wheelchair or bedbound, had cognitive impairment sufficient to prevent written informed consent, or were enrolled in another research study. Potential recruits were allowed to use walking aids. Full details of the cohort recruitment process have been published previously [13].

### Ethics Statement

Ethics approval was obtained from the National Research Ethics Service Tayside Committee on Medical Research Ethics A (ref 09/S1401/57), and written informed consent was obtained from all participants. The study was performed according to the principles of the Declaration of Helsinki [14].

### Outcomes Measurement

Physical activity was measured using the RT3 triaxial accelerometer (Stayhealthy Inc, USA). Activity counts were recorded each minute for a 7 day period. Participants were instructed to wear the accelerometer on their waistband, placed anteriorly over the left hip. Participants were instructed to start wearing the accelerometer when first rising in the morning, and to stop wearing it on retiring to bed in the evening. Accelerometers were not worn overnight for reasons of comfort and a lack of a convenient hem or belt on most nightwear to attach the accelerometer to. 24 hour periods were taken from midnight each day; the first and last day readings were discarded as they did not represent a complete 24 hour period. The RT3 accelerometer has previously been validated in older people including those using walking aids [15], can distinguish walking from sedentary activity [16], and is responsive to interventions to increase activity levels [17], [18]. Correlation between mean 24 hour counts and a trimmed measure (derived minutes of walking [16]) has previously been shown to be very high (r = 0.97, p<0.001) in this cohort [13], thus no form of trimming was applied to the count data.

Weather conditions for each day were taken from publicly accessible meteorology records at Mylnefield weather station, sited at the Scottish Crop Research Institute and contributing data via the UK Meteorological Office (www.metoffice.gov.uk). All participants were resident within 30 miles of this station. Data on hours of sunshine per day, millimetres of rain per day, maximum and minimum temperatures were collected. Maximum temperature was defined as the maximum temperature recorded in the shade for each 24 hour period (9 am to 9 am). Minimum temperature was similarly defined as the minimum temperature recorded in a frost-protected enclosure for each 24 hour period (9 am to 9 am). Presence of snow was recorded as depth of cover in centimetres, recorded at 9 am each day. Day length, defined as time between sunrise and sunset, was calculated from standard ephemerides for latitude 56.5 degrees north (the latitude of the major population centre for the cohort).

Geographical data were collected by ascertaining Global Positioning System (GPS) coordinates for each participant’s place of residence. Coordinates were then mapped onto GIS (Geographical Information Systems) to derive indices for environmental variables through a spatial join operation. The amount of nearby green space was measured in percentage of green space in the census ward (on average 5000 people); data were obtained from http://cresh.org.uk [19]. The six category Scottish Government Urban–Rural Classification 2009–2010 [20] was used to classify place of dwelling as urban or rural. The six-item classification was condensed into two types. Urban dwelling was defined as those in Large Urban Areas, Other Urban Areas, Accessible Small Towns, Remote Small Towns; rural dwelling as living in Accessible Rural and Remote Rural areas. Deprivation was measured using the Scottish Index of Multiple Deprivation (SIMD) score (http://www.isdscotland.org). This index assigns a decile ranking to each data zone in Scotland (on average 750 people) [21], based on a composite measure of 38 indicators across domains of income, employment, health, education, skills and training, housing, accessibility and crime. Lower SIMD deciles are more deprived.

Depression was measured using Hospital Anxiety and Depression score (HADS) depression subscale, to isolate depressive symptoms from somatic symptoms caused by frequent comorbid disease in this older cohort. Higher scores (maximum 21 for each subscale) denote more severe symptoms. The SF-36 was collected as a general health status tool [22]. Social capital was estimated from the ‘number of people you can turn to within walking distance’ from the Social Capital questionnaire [23]; we have previously found that these responses correlate with physical activity within the cohort [10]. Perceived Behavioural Control was measured using principles of the Theory of Planned Behaviour [24] and self-reported function was collected using the physical and psychosocial subscales of the Functional Limitations Profile (FLP) [25] (a UK version of the Sickness Impact Profile). Higher scores denote worse function (maximum 409 for physical subscale and 462 for psychosocial subscale). Self-reported comorbid disease was collected from the Older Peoples Activity Limitation (OPAL) questionnaire [26]. Categories extracted were arthritis (osteoarthritis or rheumatoid arthritis), cancer, Parkinson’s disease or other neurological disorder, diabetes, or heart disease (‘a heart condition, angina or heart attack’).

### Statistical Analysis

Univariate correlations between weather variables and activity counts were performed, with predetermined subgroup analyses being performed for those in the younger (65–80 years) and older (80+ years) subgroups. All weather variables were analysed as continuous data with the exception of snow cover. The absence of snow cover was associated with significantly higher mean activity counts than the presence of snow cover, with no difference in mean counts with higher or lower snow cover. Snow cover was thus dichotomised as present or absent. Number of people available to turn to was log-transformed prior to inclusion in the models to meet the assumptions of normality. Perceived behavioural control scores were bimodal in distribution and were therefore dichotomised into high and low perceived behavioural control (cutoff 4.1, range 1 to 6, with higher scores denoting higher control).

Weather variables (Hosmer-Lemeshow test p<0.3 on univariate analysis) were then entered into a multilevel model, incorporating participant level data at level 1, and daily weather data and counts at level 2. Analyses were conducted using the mixed modelling function of SAS v9.2. Additional variables known from previous analyses [13] to associate with activity counts in this cohort were then added to the model, along with data on comorbid disease derived from the OPAL questionnaire.

Preplanned interaction analyses were undertaken using linear mixed modelling in SAS to test the effect of the following variables on modifying the relationship between weather and physical activity: urban versus rural location, perceived behavioural control, social capital, physical function (FLP), anxiety and depression scores and comorbid disease in order to test the modifying influence of these variables on the effect of weather conditions. For each interaction analysis, the model incorporated the weather term and the effect-modifying variable, prior to fitting the interaction term.

## Results

**Figure 1:**
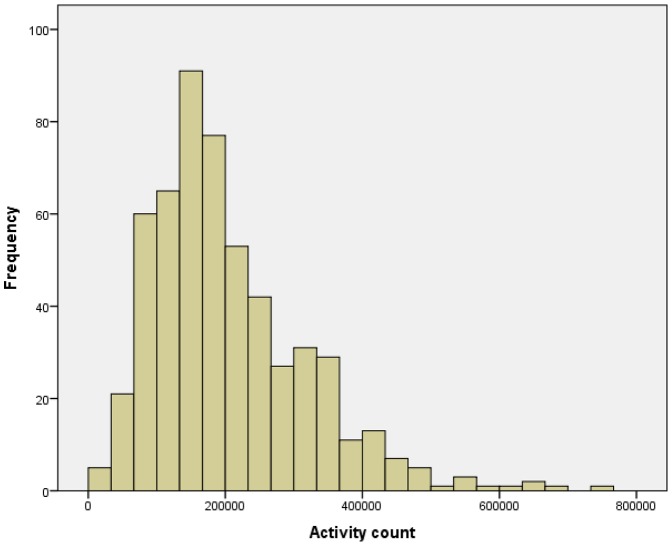
Histogram depicting distribution of mean 24(n = 547).

**Figure 2:**
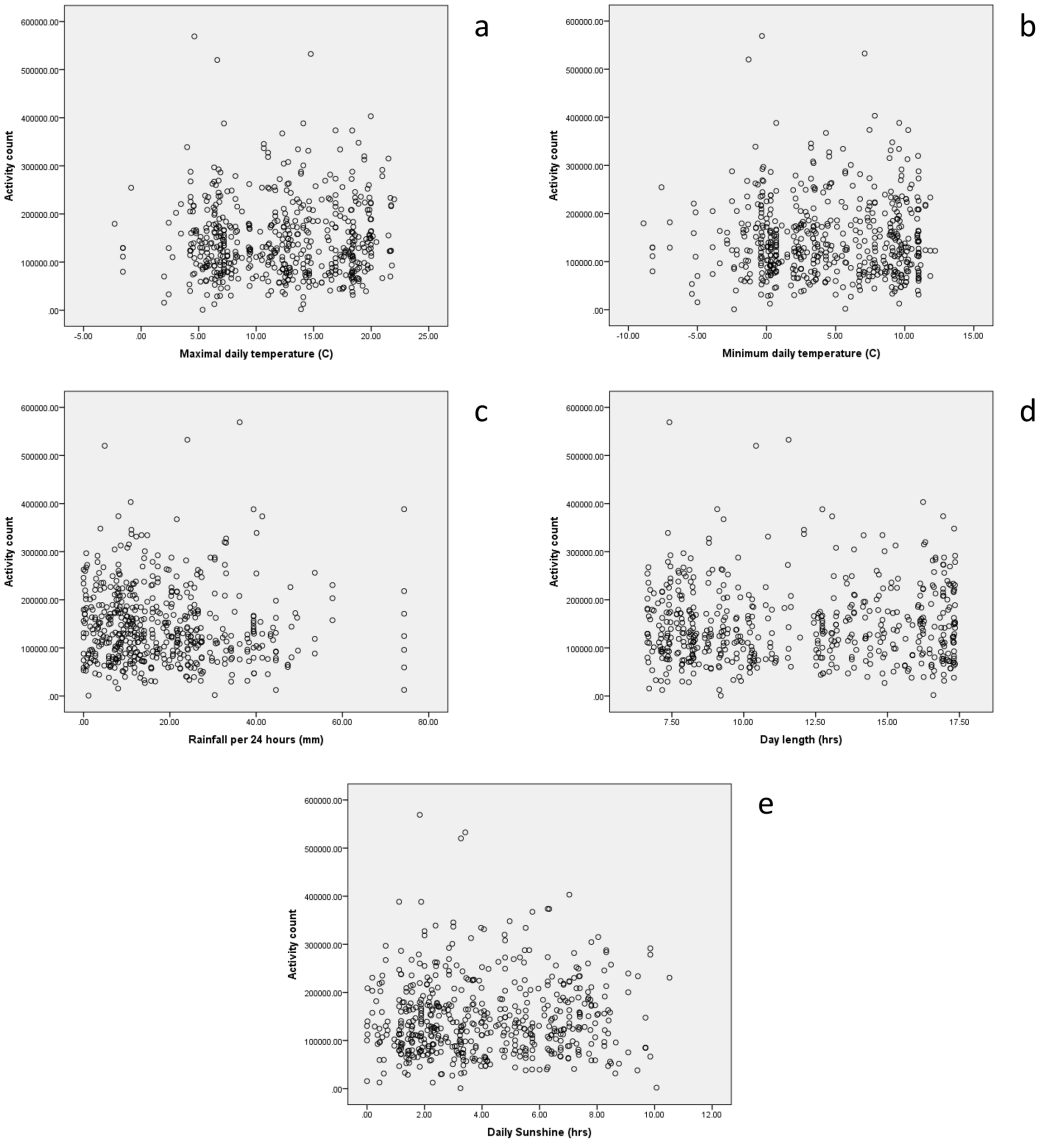
**a.** Scatter plot depicting relationship between 24(n = 3228). **b.** Scatter plot depicting relationship between 24 hour activity count and minimum daily temperature (n = 3228). **c.** Scatter plot depicting relationship between 24 hour activity count and daily rainfall total (n = 3228). **d.** Scatter plot depicting relationship between 24 hour activity count and day length (n = 3228). **e.** Scatter plot depicting relationship between 24 hour activity count and daily sunshine total (n = 3228).

Activity data were available for 547 participants, who are included in this analysis. Baseline details are given in Table 1; the mean age was 78.4 years, with a range of 65 to 105 years. Figure 1 shows the distribution of mean daily activity counts. Based on 547 participants with an expected 6 complete 24 hour data periods each (3282 days of data), data were available on 3228/3282 (98.4%) of days. Table 2 shows the univariate associations between weather variables and activity counts, further illustrated by scatterplots in Figure 2; day length and minimum temperature showed a significant positive association with daily activity counts and were therefore selected as variables to go forward into multivariable mixed models. Mean activity counts on weekend days were very similar to counts on weekdays (mean 148559 vs 146184, p = 0.43); weekday vs weekend activity was therefore not included as a separate variable in models.

**Table 1:** Baseline data (n = 547).

	Mean (SD) or proportion	Median (25^th^, 75^th^ percentile)	Range (min, max)
Mean Activity level (counts/24 hrs)	146 197 (79 655)	129521 (88253, 185037)	870, 569047
Mean Age	78.5 (7.7)	79 (72, 84)	65, 105
Male Sex (%)	250/547 (45.7)	–	–
Mean SIMD decile	5.2 (2.7)	5 (3, 7)	1,10
HADS depression score	3.7 (2.7)	3 (2, 5)	0,14
HADS anxiety score	4.2 (3.2)	4 (2, 6)	0,19
Perceived Behavioural Control score	4.87 (1.03)	5 (4.5, 5.5)	1,6
Urban dwellers (%)	173 (32)	–	–
% Green space in vicinity	63 (30)	74 (33, 94)	12, 95
**Comorbid disease:**			
Arthritis (%)	140/547 (25.6)	–	–
Neurological disorder (%)	10/547 (1.8)	–	–
Diabetes (%)	60/547 (11.0)	–	–
Heart disease (%)	179/547 (32.7)	–	–
Cancer (%)	31/547 (5.7)	–	–
**Mean SF-36 score domains:**			
Physical functioning	71.6 (23.0)	80 (55, 90)	5, 100
Role – Physical	82.5 (25.8)	100 (69, 100)	0, 100
Bodily pain	69.1 (26.7)	72 (51, 100)	0, 100
General health	67.6 (21.0)	72 (52, 85)	5, 100
Vitality	59.9 (20.7)	63 (50, 75)	0, 100
Social Functioning	88.3 (22.5)	100 (88, 100)	0, 100
Role – Emotional	96.6 (11.5)	100 (100, 100)	25, 100
Mental Health	82.8 (13.4)	85 (80, 90)	25, 100
Mean number of people you can turn to	3.1 (3.9)	2 (1, 4)	0, 50
Mean FLP physical subscore	175 (119)	192 (67, 287)	0, 409
Mean FLP psychosocial subscore	188 (115)	180 (91, 272)	0, 462
Mean daily sunshine (hrs)	4.1 (2.5)	3.6 (2.0, 6.3)	0, 10.5
Mean rainfall per 24 hours (mm)	18 (14)	14 (8, 26)	0, 74.0
Mean day length (hrs)	11.7 (3.6)	10.9 (8.2, 15.3)	6.6, 17.4
Mean of maximal daily temperature (°C)	12.0 (5.4)	12.4 (6.9, 16.9)	−2.3, 22.0
Mean of minimum daily temperature (°C)	4.4 (4.6)	4.1 (0.3, 9.0)	−8.9, 12.2

FLP: Functional Limitations Profile.

HADS: Hospital Anxiety and Depression Score.

SIMD: Scottish Index of Multiple Deprivation.

**Table 2:** Univariate associations between weather parameters and activity counts/24 hrs.

	Difference in activity counts per unit change (95% CI)				
	All participants	65–80 yrs,SIMD 1–4	65–80 yrs,SIMD 5–10	80+ yrs, SIMD 1–4	80+ yrs, SIMD 5–10
**Day length (hours)**	2081* (291,3870)	−238(−3636, 3159)	3826(248, 7405)	2823 (1.41, 5645)	3129 (−919, 7178)
**Daily Sun (hours)**	−238 (−855, 380)	−149(−1457, 1159)	−513(−1924, 897)	−629 (−1635, 377)	140 (−866, 1145)
**Minimum daily temperature (°C)**	1254** (536, 1972)	2079(679, 3479)	321(−1202, 1844)	1483 (380, 2585)	1268 (−164, 2700)
**Maximum daily temperature (°C)**	116 (−638, 870)	118(−1323, 1559 )	−139(−1767, 1489)	1351 (171, 2531)	−463 (−1950, 1024)
**Daily Rain (mm)**	−124 (−539, 291)	−352(−1060, 357)	−253(−1302, 796)	1038 (309, 1767)	−308 (−1059, 443)
**Presence of snow (present vs absent)**	50 (−15123, 15223 )	16,482(−9536, 42501)	5607(−30251, 41465)	−26416(−47440, −5391)	−7646 (−46851, 31558)

* p<0.05;

** p<0.01.

Table 3 gives the result of the overall mixed models, including age and SIMD category as covariates in the full model, but also showing models run for each age/deprivation strata separately. Of the comorbid disease categories listed in Table 1, only neurological disease, heart disease and diabetes showed significant univariate associations with weather variables; hence only these comorbidities are included in the mixed models. In these mixed models, the weather variables remained as independent associates of daily activity, along with perceived behavioural control, age and number of people the participant could turn to. Interestingly the magnitude of the effects of the weather variables were not influenced by adjustment for the other variables. For each degree increase in minimum temperature, daily activity counts increased by approximately 0.9% (1377/146497). Similarly for each hour increase in day length, daily activity counts increased by approximately 1.5% (2210/146497). This equates to a 16.5% difference between the longest day and shortest day at the latitude at which the study was conducted. On entering both minimum daily temperature and day length into mixed models, only minimum daily temperature remained in the mixed model as a significant independent associate of activity levels, along with age, perceived behavioural control, SF-36 physical function score and number of people to turn to.

**Table 3:** Mixed models: Independent predictors of daily activity counts, incorporating 1) minimum daily temperature and 2) day length, as predictors.

Variables	Difference in activity counts per unit change (95% CI)				
	All participants	65–80 yrs, SIMD 1–4	65–80 yrs, SIMD 5–10	80+ yrs, SIMD 1–4	80+ yrs, SIMD 5–10
**Model 1**					
**Minimum daily temperature (per 1°C increase)**	1319** (626, 2012)	1978** (637, 3318)	797 (−695, 2289)	1216* (113, 2319)	1481* (94, 2868)
**Age (per year increase)**	−2152** (−3763, −542)	−1336 (−4768, 2096)	−291 (−5488, 4905)	−1738 (−5465, 1989)	−5026* (−8979, −1073)
**Perceived behavioural control (high vs low)**	135689* (14360, 257018)	383661** (35975, 731348)	175402 (−259434, 610237)	−168260 (−558769, 222250)	105544 (−411386, 522474)
**SF-36 Physical functioning (per point**	1165** (852, 1478)	967** (310, 1623)	1893** (1053, 2734)	565* (81, 1049)	1468** (941, 1996)
**Number of people you can turn to (per extra person)**	17050 (−1468, 35568)	55017** (17089, 92946)	9718 (−27212, 46647)	−11848 (−44919, 21222)	−5218 (−42310, 31874)
**Neurological disorder**	−26056 (−67474, 15362)	−14033 (−119332, 91266)	−6965 (−91288, 77358)	−48196 (−118498, 22106)	−49657 (−119535, 20220)
**Diabetes**	−14290 (−32282, 3701)	−5151 (−42392, 32089)	−3787 (−46131, 38557)	−18691 (−44024, 6641)	−22706 (−61825, 16412)
**Heart disease**	−3614 (−15748, 8519)	1910 (−24162, 27983)	−12345 (−38050, 13361)	5228 (−16612, 27068)	−4715 (−26663, 17232)
**Model 2**					
**Day length (per additional hour)**	1931* (264, 3598)	198 (−2854, 3251)	4533** (1116, 7951)	2048 (−796, 4891)	2846 (−1244, 6936)
**Age (per year increase)**	−2903** (−4584, −1224)	−1337 (−4867, 2192)	−3488 (−8759, 1782)	−2147 (−5992, 1698)	−6716** (−11125, −2307)
**Perceived behavioural control (high vs low)**	138182* (10999, 265365)	455611* (102021, 809201)	30334 (−423285, 483953)	−181803 (−585633, 222026)	−26445 (−601669, 548778)
**Number of people you can turn to (per extra person)**	17189 (−2250, 36627)	55197** (16489, 93906)	15801 (−22886, 54489)	−10503 (−44635, 23629)	−11095 (−52447, 30256)
**Neurological disorder**	−42431 (−85667, 806)	−32804 (−138924, 74755)	−52737 (−139023, 33549)	−55843 (−128341, 16656)	−48969 (−129176, 31238)
**Diabetes**	−22769* (−41516, −4023)	−16483 (−53753, 20787)	−27022 (−70375, 16332)	−19037 (−45422, 7348)	−28506 (−72131, 15119)
**Heart disease**	−10093 (−22637, 2451)	−5044 (−30672, 20584)	−21991 (−48396, 4414)	1423 (−20982, 23827)	−6862 (−31373, 17650)

* p<0.05.

** p<0.01.

Table 4 reports the results of tests of interaction between a range of psychosocial and environmental factors and the weather variables on their association with activity counts. Age, perceived behavioural control, anxiety, depression or number of people to turn to did not modify the effect of either day length or minimum temperature on daily activity, and medical conditions (diabetes, heart disease and neurological disease) which might be expected to have an association with physical activity did not modify the effect of weather variables on activity levels. The only significant interactions were between urban versus rural dwelling and day length, and between social functioning scores on the SF-36 and day length. Urban dwelling participants showed a less strong association between day length and activity levels, as did participants with lower social functioning scores.

**Table 4:** Interaction effects between weather variables and moderator variables on daily activity levels.

Interaction term	t	p	Interaction term	t	p
Min daily temperature × Age	−0.02	0.98	Day length × Age	0.67	0.51
Min daily temperature × Perceived behavioural control	1.09	0.28	Day length × Perceived behavioural control	0.45	0.66
Min daily temperature × Urban – rural	−0.38	0.71	Day length × Urban – rural	−2.01	0.04
Min daily temperature × No. of people you can turn to	0.36	0.72	Day length × No. of people you can turn to	0.31	0.75
Min daily temperature × HADS Anxiety	0.16	0.87	Day length × HADS Anxiety	0.01	0.99
Min daily temperature × HADS Depression	0.37	0.71	Day length × HADS Depression	−0.32	0.75
SF-36 domains:					
Min daily temperature × Physical Functioning	1.53	0.13	Day length × Physical Functioning	1.55	0.12
Min daily temperature × Role - Physical	0.66	0.51	Day length × Role - Physical	1.55	0.12
Min daily temperature × Bodily Pain	0.98	0.33	Day length × Bodily Pain	−0.23	0.82
Min daily temperature × General Health	1.45	0.15	Day length × General Health	1.89	0.06
Min daily temperature × Vitality	−0.03	0.97	Day length × Vitality	0.67	0.50
Min daily temperature × Social Functioning	1.06	0.29	Day length × Social Functioning	2.16	0.03
Min daily temperature × Role - Emotional	−0.04	0.97	Day length × Role - Emotional	1.62	0.11
Min daily temperature × Mental Health	1.14	0.25	Day length × Mental Health	1.76	0.08
Min daily temperature × Diabetes	−0.55	0.58	Day length × Diabetes	−0.39	0.70
Min daily temperature × Neurological disease	−1.48	0.14	Day length × Neurological disease	−0.02	0.98
Min daily temperature × Heart disease	−0.99	0.32	Day length × Heart disease	0.71	0.48

HADS: Hospital Anxiety and Depression Score.

SF-36: Short form 36 questionnaire.

## Discussion

This study has found a modest but noteworthy relationship between weather variables and physical activity in this cohort of community-dwelling older people in Scotland. The observed associations were not modified by inclusion of variables describing social support or perceived behavioural control – key modifiable predictors of activity in this cohort; but a weak modifying effect was noted for social functioning and urban versus rural dwelling place on the association between weather variables and activity.

Previous work has previously demonstrated a strong relationship between both day length and weather in a smaller, more highly selected cohort of frail older people resident in Scotland [9]; in this previous study, activity varied twofold between summer and winter. The magnitude of effect seen in the current study was much smaller, with a 16.5% summer to winter difference. This could be due to differences in the characteristics of the current cohort; they were less highly selected by dint of our observational design with broad-based sampling, rather than a randomised controlled trial with exclusion criteria, they were also less functionally impaired and more active than in the previous study. As a more robust population, they may have been less affected by either falls or by the fear of falling, which may have contributed to the smaller difference between winter (with its higher risk of ice and snow) and summer activity levels. In children, younger adults and healthy older Japanese adults, rain has been shown to have significant negative effects on activity levels [6], although this finding was not borne out in the current study. Previous studies have shown slightly lower variation in activity levels with day length; these studies were performed at lower latitudes and hence had less extreme differences in day length between seasons, which may explain the smaller variability (10% and 13%) in activity between highest and lowest activity levels [27], [28].

The utility of testing associations between weather and activity lies in finding ways to ameliorate any deleterious effect of inclement weather on activity levels. Finding ways to do this could help future physical activity enhancing interventions to overcome the barriers posed by inclement weather; previous studies have suggested that inclement weather is a perceived barrier to activity in young to middle-aged adult populations, and that provision of indoor leisure facilities could potentially overcome some of the effects of inclement weather. Providing physical protection against inclement weather, or choosing activities that are not influenced by weather, provide the obvious ways of approaching this issue. An alternative strategy is to bolster personal physical and psychosocial attributes that might buffer the effect of adverse weather conditions. Unfortunately, we found no evidence from this study that such attributes were able to overcome the barrier of inclement weather – neither local geography, perceived behavioural control nor anxiety and depression status modified the influence of weather conditions on physical activity.

Strengths of the study approach include the range of ages, activity levels, social and physical and environments included in our sample, the use of objective activity recording, our ability to marry daily activity data with weather data from the same day over a period of several days, and the availability of other environmental, social and psychological variables for our cohort. The limited follow-up time for individuals within the study limits our ability to test the effect of changes in season on activity within individuals, and the study was dependent on participants volunteering to take part, which inevitably leads to a degree of selection bias. The study site has relatively cool weather with frequent precipitation and large variability in day length; results from hotter locations or locations with less day length variability [29] may show different effects of weather variables, e.g. less activity in hotter weather [30]. We are not able to distinguish indoor from outdoor activity; objective measurement of this distinction would be of considerable value in future studies, and would give further insight into the relationships between weather and different types of physical activity. Although activity counts are not an intuitive metric for assessing physical activity, the relation between activity counts and energy expenditure in metabolic equivalents is prone to error [31], and has not been validated in older people. We have not therefore translated our results into metabolic equivalents, and percentage changes in activity levels may provide an easier way of conveying the impact that factors such as weather conditions have on activity levels. Our model-building approach in this analysis was data-driven, rather than being based on *a priori* assumptions as to which factors would be associated with activity levels. We are thus unable to comment on whether similar relationships would hold for other cohorts, and replication of the associations that we have found in independent cohorts would therefore be valuable.

In conclusion, we have shown that minimum temperature and day length variables influence objectively measured physical activity levels in a large random sample of older people. Little effect of moderator variables was seen, suggesting that ameliorating the deleterious effects of inclement weather on activity levels is unlikely to be achieved by treating anxiety and depression, enhancing social networks or enhancing psychological variables such as perceived behavioural control. Subjective general health and physical function showed weak, non-significant interactions with weather variables, however given that enhancing physical activity is one of the major ways to improve both of these domains, this observation may not translate easily into interventions. It suggests that good physical health ameliorates the effect of bad weather – but more physical activity would be needed to boost physical health to get this effect. Ameliorating the effects of inclement weather on physical activity may therefore require direct protection from the elements (e.g. taxi or public transport to venues where leisure time activity occurs), or the design of activity promoting programmes that use activities less influenced by weather (e.g. indoor activities). Future studies could thus usefully examine the influence of the built environment, the factors contributing to rural vs urban activity, and the choice of leisure activities on the interaction between weather and physical activity in older people.
